# Adjuvant Therapy in “Intermediate-Risk” Early-Stage Cervical Cancer: To Treat or Not to Treat? Systematic Review and Meta-Analysis

**DOI:** 10.3390/cancers17081320

**Published:** 2025-04-14

**Authors:** Chiara Ripepi, Francesco Cracco, Giuseppe Ricci, Luigi Nappi, Stefano Restaino, Giuseppe Vizzielli, Stefania Carlucci, Guglielmo Stabile

**Affiliations:** 1 UOC Clinica Ostetrica e Ginecologica, Department of Medicine, Surgery and Health Sciences, University of Trieste, 34137 Trieste, Italy; chiara.ripepi@burlo.trieste.it (C.R.); francesco.cracco@sacrocuore.it (F.C.); giuseppe.ricci@burlo.trieste.it (G.R.); 2 Institute for Maternal and Child Health IRCCS “Burlo Garofolo”, 34137 Trieste, Italy; 3 Department of Medical and Surgical Sciences, Institute of Obstetrics and Gynecology, University of Foggia, 71121 Foggia, Italy; luigi.nappi@unifg.it; 4 Department of Maternal and Child Health, Obstetrics and Gynecology Clinic, University-Hospital of Udine, 33100 Udine, Italy; stefano.restaino@asufc.sanita.fvg.it; 5 Department of Medicine, Obstetrics and Gynecology Clinic, University of Udine, 33100 Udine, Italy; giuseppe.vizzielli@uniud.it; 6 Department of Clinical and Experimental Medicine, Institute of Obstetrics and Gynecology, University of Foggia, 71122 Foggia, Italy; s.carlucci86@gmail.com

**Keywords:** intermediate-risk early-stage cervical cancer, ajuvant therapy, ater surgery treatment, surgery, radiotherapy

## Abstract

International guidelines do not take a clear position regarding the management of patients with “intermediate-risk” cervical cancer, and they remain noncommittal on the necessity of adjuvant therapy following radical hysterectomy. To determine if adjuvant therapy after surgery adds benefit in “intermediate-risk” cervical cancer patients in terms of recurrence rate and mortality rate, we performed a systematic review with meta-analysis on Google Scholar, PubMed, and Scopus. The inclusion criteria were studies with >100 patients with intermediate-risk cervical cancer according to the Sedlis criteria, RCTs, or propensity score-matched cohort studies reporting oncological outcomes and treatment-related toxicity, comparing a surgery-only cohort with an adjuvant therapy cohort. We found 11 studies that met our inclusion criteria, comprising a total of 4011 patients. The mean follow-up was 80.7 months. No statistically significant differences were found for both outcomes, respectively, recurrence rate OR 0.92 (95% CI 0.70–1.20, I^2^ 38%) and mortality rate OR 1.05 (95% CI 0.77–1.46, I^2^ 41%). However, a statistically significant difference was found in the site of recurrence–pelvic OR 0.48 (95% CI 0.23–0.98, *p* < 0.001, I^2^ 42%) or distant OR 2.10 (95% CI 1.02–4.33, *p* < 0.001 I^2^ 42%). Treatment-related risk of Grade 3 or 4 toxicity was statistically significant. Our meta-analysis shows that oncological outcomes in this class of patients are similar between the two groups. However, while awaiting the results of new RCTs, it is crucial to define a personalized diagnostic and therapeutic strategy for each patient.

## Simple Summary

To date, international guidelines do not take a clear position regarding the management of patients with “intermediate-risk” cervical cancer, and they remain noncommittal on the necessity of adjuvant therapy following radical hysterectomy. For this reason, we performed a systematic review with meta-analysis to determine if adjuvant therapy after surgery adds benefit in “intermediate-risk” cervical cancer patients in terms of recurrence rate and mortality rate. We found 11 studies that met our inclusion criteria, comprising a total of 4011 patients. No statistically significant differences were found for both outcomes, respectively, recurrence rate and mortality rate. A statistically significant difference was found in the site of recurrence—pelvic or distant. Our meta-analysis shows that oncological outcomes in this class of patients are similar between the two groups. However, while awaiting the results of new randomized controlled trials (RCTs), it is crucial to define a personalized diagnostic and therapeutic strategy.

## 1. Introduction

To date, the treatment of choice for early-stage cervical cancer is surgery, consisting of radical hysterectomy with bilateral salpingo-oophorectomy and pelvic lymphadenectomy or bilateral sentinel lymph node sampling [1]. However, the role of adjuvant therapy remains controversial and should be personalized based on histological characteristics from the final pathological analysis [2]. Based on pathological findings that correlate with different risks of recurrence, early-stage cervical cancers are classified into three risk categories: low, intermediate, and high [2]. “Intermediate-risk” cervical carcinomas are characterized by negative lymph nodes, negative margins and parametria, and the presence of at least two risk factors: tumor size ≥ 2 cm, lymphovascular space invasion (LVSI+), and deep stromal invasion > 1/3 [3]. The combination of these risk factors defines the so-called “Sedlis Criteria,” established by the GOG 92 trial published in 1999, which predicted a 3-year recurrence rate of ≥30% [4]. Although the results of this trial support adjuvant radiotherapy after treatment, numerous other studies have since been published on this topic, fueling ongoing debate. In this context, we decided to conduct this meta-analysis to clarify the current evidence, pending the publication of new trial results.

## 2. Materials and Methods

This research was approved by the Institutional Review Board of the Institute for Maternal and Child Heath IRCCS Burlo Garofolo (RC 08/2022). A literature search was carried out in October 2024 using different combinations of the following terms: (Intermediate Risk) AND [(Cervical Neoplasm) OR (Cervical Cancer) OR (Cervical Tumor) OR (Cervical Carcinoma) OR (Cervix Neoplasm) OR (Cervix Cancer) OR (Cervix Tumor) OR (Cervix Carcinoma)] AND [(Postoperative Therapy) OR (After Surgery Therapy) OR (Adjuvant Therapy) OR (Adjuvant Radio Therapy)] AND (Hysterectomy). Articles published from 1999 to September 2024 were obtained from Google Scholar, PUBMED and Scopus. Inclusion criteria for articles in our meta-analysis were as follows: (1) at least 100 patients; (2) patients diagnosed with “intermediate-risk” cervical cancer according to Sedlis’ criteria that underwent primary radical hysterectomy involving lymphadenectomy or bilateral sentinel node sampling; (3) no further treatment or radiotherapy (RT) or concurrent chemo-radiotherapy (CCRT) was given after radical hysterectomy; and (4) reported relevant outcomes, such as total recurrence, local recurrence, distant recurrence, mortality, overall survival (OS), disease-free survival (DFS), and treatment-related toxicity. In our study, we included randomized controlled, observational prospective cohort, retrospective cohort, and case-control studies. Studies were excluded if (1) full-text was not available; (2) necessary data could not be extracted; (3) they had a single-arm cohort design; (4) they were not published in English; or (5) they failed to score adequately in the quality assessment.

All studies identified were examined for year, citation, title, authors, abstract, and their full texts. Two researchers (C.R. and G.S.) manually screened the studies for duplicates, which were then removed. The PRISMA guidelines were followed [5].

**Figure 1:**
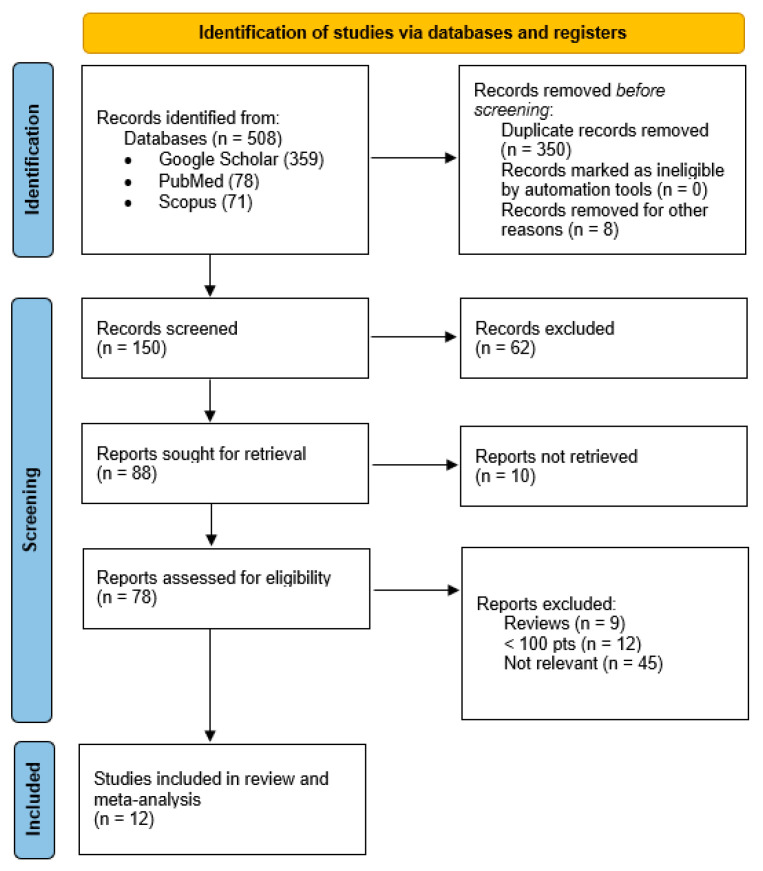
PRISMA flow diagram.

The PRISMA flow diagram of the selection process is provided in Figure 1. For the eligibility process, two authors (C.R. and G.S.) independently screened titles and abstracts of all non-duplicated papers and excluded those not pertinent to the topic. The same two authors independently reviewed the full text of papers that passed the first screening. They identified those to be included in the review by extracting the following information: authors’ name, publication year, study design, sample size, 2009 FIGO stage [6], cancer histology, tumor size, deep stromal invasion, lymph vascular space invasion, type of adjuvant therapy, recurrence rates (total, local, and distant), survival rates (mortality, OS, and DFS), treatment-related toxicity, and follow-up. Discrepancies were resolved by consensus. The methodological quality and risk of bias of the included studies was assessed using the Newcastle–Ottawa scale for non-randomized studies and modified Jadad score scale for randomized controlled trials (Supplementary Tables S1 and S2).

### Statistical Analysis

Statistical analyses were performed using R Studio version 4.4.1, and results associated with *p* < 0.05 are considered significant. We calculated pooled odds ratios (ORs) and associated 95% confidence intervals (CIs) using a random effects model and the DerSimonian–Laird method or Mantel–Haenszel method. Heterogeneity of outcomes was assessed based on I^2^ and visual analysis of forest plots. We considered I^2^ > 50% as high heterogeneity, in which case we conducted subgroup and sensitivity analyses to obtain more detailed insights and to assess potential heterogeneity sources. Sensitivity analyses were performed by removing one study at a time and repeating the meta-analysis. The percentage of total recurrence is calculated based on the entire cohort, while the percentage for the specific site of recurrence is calculated only among patients who experienced a recurrence.

## 3. Results

Twelve studies were included in the meta-analysis, of which two were RCTs, while nine were retrospective cohort studies, for a total of 4011 patients. Only 277 out of 4011 patients come from RCT. Since the trial by Rotman et al. [7] represents a continuation of the follow-up of the original Sedlis et al. trial, the patient cohort was counted only once (see Table 1). Seven articles assessed survival and mortality rates, while all eleven studies evaluated disease-free survival and recurrence rates. All studies had cohorts with over 100 patients, ranging from 134 to 960. The FIGO 2009 staging system [6], the most used across the studies, was considered: 3492 patients were in stage IB (87.1%), while 519 were in stage IIA (12.9%); all patients included had negative lymph nodes. Among 1723 patients, histology was reported, with 1267 having squamous cell carcinoma (73.5%) and 456 (26.5%) with non-squamous histology, including adenocarcinomas, adenosquamous, and other types.

Regarding treatment cohorts, only studies comparing a “surgery-only” (SO) cohort and a “surgery + adjuvant therapy” (ADJ) cohort were considered, excluding studies involving neoadjuvant therapy. In retrospective studies, the decision to administer adjuvant treatment and the type of treatment was at the discretion of the center and the physician. This identified 1599 patients (39.9%) in the surgery-only cohort and 2412 patients (60.1%) in the surgery + adjuvant therapy cohort. Adjuvant therapies across studies varied, but they primarily included radiotherapy (45–60 Gy) for 1732 patients (71.8%) and concomitant chemoradiotherapy for 680 patients (28.2%). The “Sedlis criteria” were used to categorize patients in all studies; however, these criteria were not consistently specified in some studies (Table 1).

For tumor size, most patients fell within the “2–4 cm” range, representing 60.9% of patients in the surgery-only cohort and 58.0% in the surgery + adjuvant radiotherapy cohort. Only the cohort by Cao et al. [12] had the most patients with tumor sizes ≥ 4 cm, equally distributed across the three cohorts. Lymphovascular space invasion was present in the majority of patients, with a notable imbalance in the surgery-only group in the Sedlis trial. When specified, Category 1 (LVSI+, DSI deep 1/3, and any size) was the most represented category in both the surgery-only and surgery + adjuvant therapy cohorts. In contrast, Category 4 (LVSI+, DSI superficial 1/3, and ≥5 cm) was the least represented, with only two patients in the surgery group and eight in the surgery + adjuvant group. The mean follow-up was 80.7 months.

### 3.1. Recurrence

**Figure 2:**
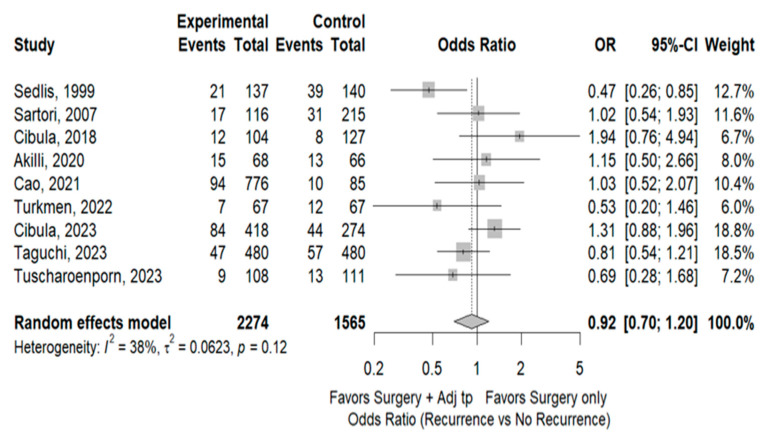
Total recurrence rate. References: [3,4,8,10,11,12,14,15,16].

**Figure 3:**
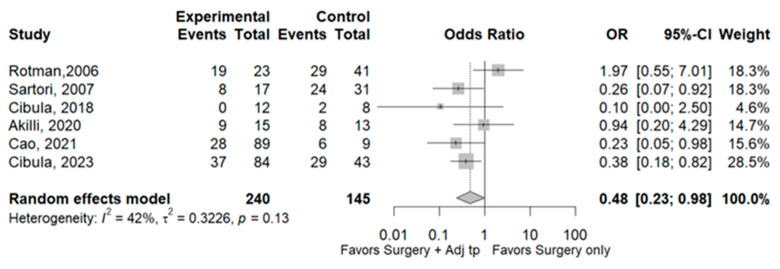
Local recurrences. References: [3,7,8,10,11,12].

**Figure 4:**
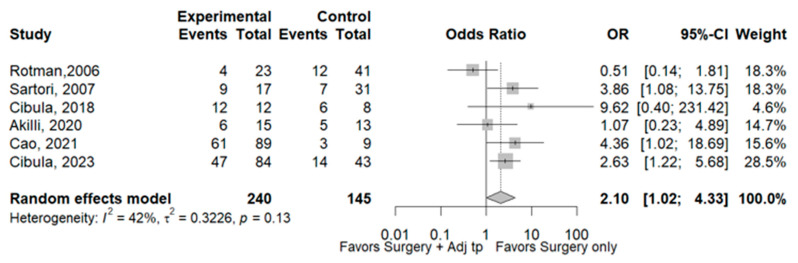
Distant recurrences. References: [3,7,8,10,11,12].

Eleven studies [3,4,7,8,9,10,11,12,14,15,16] including 4011 patients reported total recurrence rates for the SO group (14.3%, 229/1599) and the Adj group (12.9% 312/2412). The rates did not differ significantly between the two groups (OR 0.75, 95% CI 0.60–1.35; I^2^ = 58%). Sensitivity analyses identified two studies [7,9] as a potential source of heterogeneity. Excluding this study led to the same result as the complete meta-analysis, but with lower heterogeneity (OR 0.92, 95% CI 0.70–1.20; I^2^ = 38%; Figure 2). Six studies [3,7,8,10,11,12] including 2526 patients reported local recurrence rates for the SO group (67.6% 98/145) and Adj group (42.1% 101/240). The rates differ significantly between the two groups, with a lower rate in the ADJ group (OR 0.48, 95% CI 0.23–0.98; I^2^ = 42%; Figure 3). The same six abovementioned studies reported distant recurrence rates of 32.4% (47/145) and 57.9% (139/240), respectively. SO was associated with a significantly lower rate (OR 2.10, 95% CI 1.02–4.33; I^2^ = 42%, Figure 4). The 3-year disease-free survival is 83.5% in the SO group and 86.5% in the ADJ group (Table 2).

### 3.2. Survival

**Figure 5:**
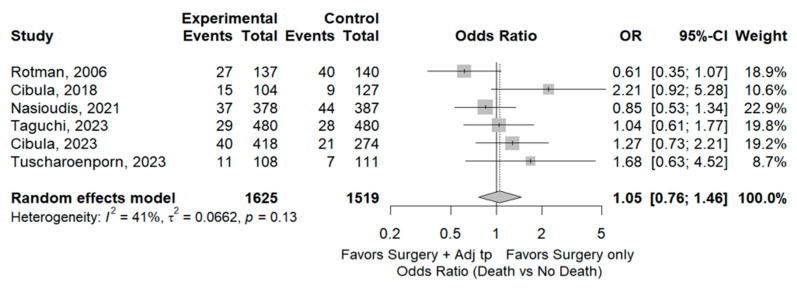
Mortality Rate. References: [3,7,10,13,15,16].

Seven studies [3,5,10,13,15,16] including 3316 patients reported mortality rates for the SO group (9.9% 153/1553) and the ADJ group (9.2% 163/1763). These rates were comparable between the two groups (OR 1.15, 95% CI 0.80–1.60; I^2^ = 65%). Sensitivity analyses identified one study [9] as a potential source of heterogeneity. Excluding this study led to the same result as the full meta-analysis, but with lower heterogeneity (OR 1.05, 95% CI 0.77–1.46, I^2^ 41%) (Figure 5).

**Figure 6:**
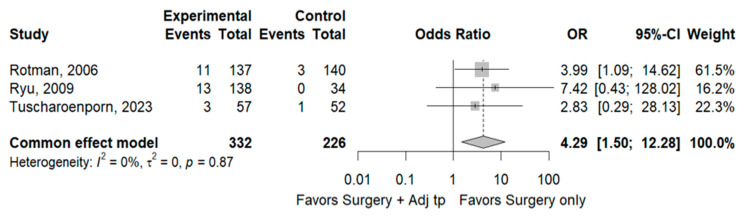
Treatment-related toxicity Grade 3 or 4. References: [7,9,16].

Overall survival at 5 years was also comparable between the two groups, at 88.9% and 89.2%, respectively. Lastly, the rate of Grade 3 or 4 treatment-related toxicity was 1.8% (4/226) and 8.1% (27/332), respectively, with a higher risk in patients undergoing adjuvant therapy after surgery (OR 4.29, 95% CI 1.50–12.25, *p* = 0.87, I^2^ 0%) (Figure 6).

## 4. Discussion

In our meta-analysis, we aimed to investigate whether there are differences in terms of recurrence and mortality rates in patients with early-stage cervical cancer classified as “intermediate-risk” based on Sedlis criteria, treated with either surgery alone or surgery plus adjuvant therapy. We did not find any significant differences in terms of total recurrence rate or mortality rate. However, as expected, in the surgery plus adjuvant therapy group, the risk of local recurrence is lower, while the risk of distant recurrence increases. Additionally, it is widely accepted that patients undergoing surgery plus adjuvant therapy experience significantly higher treatment-related toxicity.

Indeed, it is known that in patients with early cervical cancer, combining multiple treatment modalities significantly increases the incidence and severity of adverse events, including lymphedema, sexual dysfunction, urinary complications, diarrhea, constipation, and bowel obstruction [17].

We decided to include articles starting from the RCT published by Sedlis in 1999 [4], as this trial somehow established the definition of the “intermediate-risk” class, and subsequent studies adapted to this standard. Sedlis et al. are the only authors in the studies included in our meta-analysis able to demonstrate a statistically significant difference in the recurrence rate between the two groups, with a 47% reduction in risk for patients undergoing surgery plus adjuvant therapy. However, when comparing the recurrence rates of this trial with more recent studies, it is evident that the latter are much lower. For instance, Cibula et al. describe significantly better local control rates compared to the analogous arms of GOG 92, with pelvic recurrence numbers of 2 vs. 19 in the surgical arm and 0 vs. 29 in the surgery plus adjuvant therapy arm [10]. The reasons for this much better outcome in the current study could be multifactorial.

First, although patients in the GOG 92 trial were randomized to two adjuvant treatment strategies, the prevalence of risk factors was not balanced in the two arms, with a higher prevalence of LVSI (35% vs. 24%) and more patients with tumors > 4 cm in the RS group [4]. Assessment of inclusion criteria, including risk factors, reflected the clinical practice of more than 20 years ago. Indeed, it is essential to consider that in the 25 years since the publication of that trial, there has been considerable evolution in the preoperative selection of patients, intraoperative assessment, and surgical techniques. Looking at the data from the 1997 randomized prospective study by Landoni et al. [18], we see that in patients undergoing surgery, after the surgical intervention, upstaging (>pT2a) was achieved in 19% of patients with a presumed mass < 4 cm, and in 35% of patients with a mass ≥ 4 cm. However, positive margins were still present in 6% and 22% of patients, and LN+ in 25% and 31%, respectively. Nowadays, these numbers would represent a significant failure in sample selection, given the tools available to us. In fact, the sensitivity and specificity of MRI and PET/CT increased, deeming these investigations essential in the preoperative selection of patients [19]. Recently, T. L. Pan et al. published a study that compares tumor size assessment by pre-operative evaluation (physical examination and/or imaging) with tumor size on final pathology [20]. Results show that the pre-operative physical examination, MRI, ultrasound, and CT all overestimated tumor size, but statistically significant bias was observed only for physical examination (*p* < 0.0001) and MRI (*p* = 0.0102). Notably, among patients who underwent pre-operative MRI, 83.2% of those with a tumor size of 2–4 cm—as determined by MRI—showed concordance with the final pathology measurements.

In addition, a crucial role is nowadays played by sentinel lymph node sampling, which allows for detecting over 90% of N1 cases [19]. Although there are currently no precise data regarding ITCs in early-stage cervical cancer, micrometastatic pelvic lymph node involvement seems to be similar to that of patients with macrometastasis. Therefore, the detection of micrometastasis by ultrastaging of SLN, determining un upstaging of the disease from FIGO 2018 Stage I to FIGO 2018 Stage III, modifies the therapeutic approach [1]. This highlights the importance of sentinel lymph node ultrastaging for correct patient selection [21]. Looking at clinical practice, the ESGO/ESMO/ESP [1] and NCCN [22] guidelines do not clearly define the use or non-use of adjuvant therapy in this group of patients, but leave the choice to the clinician after discussion with the patient. We know that the criteria defined by Sedlis could be termed “one size fits all” since they do not consider the histology of the disease but focus solely on the factors mentioned earlier. In 2021, Levinson et al. attempted to go beyond the classic Sedlis criteria by creating specific nomograms based on histology and demonstrated that the risk factors are not common [23]. In particular, for SCC, deep stromal invasion (DSI) in the lower one-third has an HR of 7.05 (2.99–16.64), while for AC, a mass > 4 cm has an HR of 4.69 (1.25–17.63). Indeed, in the follow-up of the Sedlis trial, SCC tumors had a 28% risk of recurrence post-hysterectomy with no further treatment, which was reduced to a 20% recurrence risk after receiving adjuvant radiation. In contrast, those with AC who had no further treatment post-hysterectomy had a 44% risk of recurrence, which was considerably reduced to 9% for those patients who received adjuvant radiation [4]. This highlights how histology plays a crucial role in tumor behavior and the risk of potential recurrence.

This meta-analysis, however, demonstrates a benefit, as expected, in local control from adjuvant RT (OR 0.49, 95% CI 0.23–0.98) and confirms data reported by various studies. On the other hand, it should be noted that treatment overlapping might result in an increase in the number and severity of treatment-related morbidity affecting patients’ quality of life. Lymphedema was observed more frequently in patients who received postoperative RT than in those who did not receive RT or CT (*p* < 0.001) [24]. Ryu et al. [9] considered the rate of Grade 3–4 hematologic and gastrointestinal toxicity in the SO, surgery + RT, and surgery + concurrent chemo-radiotherapy (CCRT) groups and it was 0%, 6.1%, and 13.4%, respectively (*p* > 0.05), and all toxicities were transient and tolerable with supportive treatment. In radiotherapy-naïve patients, the treatment of choice for recurrence is pelvic irradiation combined with brachytherapy. However, if the patient has already undergone radiotherapy, the preferred option for central pelvic recurrence is pelvic exenteration, with the associated morbidity [1].

Since there are no clear guidelines regarding the adjuvant therapy to be used, several studies [25,26,27,28] considered both RT and CCRT based on optimal results in local control for patients with locally advanced cervical cancer. However, most of these studies failed to demonstrate the superiority of CCRT. Moreover, a recent meta-analysis including 16 studies with a total of 5052 patients showed that there is no statistically significant difference in recurrence and mortality between adjuvant chemotherapy (AC) and surgery + RT/CCRT [28].

Therefore, based on our findings and the results of our meta-analysis, there is no difference in recurrence rate and mortality rate between patients with stage IB2 “intermediate-risk” cervical carcinoma who underwent SO and those treated with surgery combined with any adjuvant treatment, at the expense of a significant increase in treatment-related toxicity for those receiving adjuvant therapy. Consequently, it seems advisable to avoid associating adjuvant therapy with most patients, considering the Sedlis factors as questionable and assuming that radical surgery was performed in a referral center in accordance with ESGO quality indicators [29].

The strength of our meta-analysis lies in the methodological rigor with which it was conducted, including many studies and performing various subgroup analyses. However, there are several limitations, primarily due to the retrospective nature of the studies, but especially related to sample selection. In fact, given the structure of the studies included, it is not possible to perform specific analyses by risk factors (LVSI, DOI, and histology) or by type of therapy (RT vs. CCRT vs. CHT), which represents a significant bias. Finally, we must also consider that the selected studies cover a long period of time during which RT and surgery evolved significantly.

## 5. Conclusions

The results of this meta-analysis confirm the findings from the most recent studies on the topic, namely the lack of evidence for the benefit of adjuvant therapy—whether RT, CCRT, or CT—in patients undergoing radical surgery (according to ESGO quality indicators [29]) for early-stage “intermediate-risk” cervical cancer. Furthermore, our meta-analysis highlights the need to modernize and standardize the criteria for this risk class, as they are currently relatively imprecise, especially considering advancements in techniques and knowledge. Finally, it is important to remember that, according to the new ESGO-ESTRO-ESP guidelines [1] for the management of cervical cancer, radical surgery should not be performed if an indication for adjuvant radiotherapy is known before surgery. Instead, the patient should be referred to for primary chemoradiation, and combined treatment with two modalities should be avoided. While awaiting the results of the CERVANTES trial [30], the best option may be to discuss with each patient the risks and benefits of each therapeutic option to reach a personalized decision, as well as to carry out increasingly meticulous preoperative selection, considering the “seed of doubt” raised by the results of recent studies, which seem to indicate adenocarcinoma as tricky histology.

## Figures and Tables

**Table 1:** Clinical parameters reported by the selected studies.

Study	Design	Pts (*n*)	Adjuvant Therapy	FIGO Stage (2009)	Histology	Tumor Size	Intermediate-Risk Criteria
Sedlis, 1999 [4]	RCT	277	No 140 (50.5) RT 137 (49.5)	IB	SCC: No 115 (82.1); RT 103 (75.2) Non-SCC: No 25 (17.9); RT 34 (24.8)	≤3 cm: No 58 (42.6); RT 39 (29.3) >3 cm: No 78 (57.4); RT 94 (70.7)	LVSI+, deep third, any: No 69 (49.3); RT 60 (43.8) LVSI+, middle third, ≥2 cm: No 37 (26.4); RT 28 (20.4) LVSI+, superficial third, ≥5 cm: No 0 (0.0); RT 1 (0.7) LVSI−, middle/deep third, ≥4 cm: No 34 (24.3); RT 48 (35.1)
Rotman, 2006 [7]	RCT	277	No 140 (50.5) RT 137 (49.5)	IB	SCC: No 115 (82.1); RT 103 (75.2) Non-SCC: No 25 (17.9); RT 34 (24.8)	≤3 cm: No 58 (42.6); RT 39 (29.3) >3 cm: No 78 (57.4); RT 94 (70.7)	LVSI+, deep third, any: No 69 (49.3); RT 60 (43.8) LVSI+, middle third, ≥2 cm: No 37 (26.4); RT 28 (20.4) LVSI+, superficial third, ≥5 cm: No 0 (0.0); RT 1 (0.7) LVSI−, middle/deep third, ≥4 cm: No 34 (24.3); RT 48 (35.1)
Sartori, 2007 [8]	Retrospective	331	No 215 (65.0) RT 116 (35.0)	IB	NA	NA	NA
Ryu, 2009 [9]	Retrospective	172	No 34 (19.8) RT 49 (28.5) CCRT 89 (51.7)	IB1 107 (62.2) IB2 18 (10.4) IIA 47 (27.3)	SCC 130 (75.5) Non-SCC 42 (24.5)	NA	LVSI+, deep third, any: 38 (54.3) LVSI+, middle third, ≥2 cm: 8 (11.4) LVSI+, superficial third, ≥5 cm: 1 (1.4) LVSI−, middle/deep third, ≥4 cm 23 (32.9)
Cibula, 2018 [10]	Retrospective	231	No 127 (55.0) RT 6 (2.6) CCRT 98 (42.4)	IB	SCC: No 96 (75.6); RT: 67 (64.4) Non-SCC: No 31 (24.4); RT: 37 (35.6)	≤2 cm: No 34 (26.8); 21 (20.4) 2–4 cm: No 78 (61.4); 61 (59.2) >4 cm: No 15 (11.8); 21 (20.4)	DSI: No 13.0 ± 6.3 mm; RT 13.4 ± 6.0 mm LVSI+: No 67 (52.8); RT 78 (75.0)
Akilli, 2020 [11]	Retrospective	134	No 66 (49.3) RT 68 (50.7)	IB	SCC: No 55 (83.3); RT 53 (77.9) Non-SCC: No 10 (15.2); RT 14 (20.6)	<4 cm: No 49 (74.2); RT 40 (58.8) ≥4 cm: No 17 (25.8); RT 28 (41.2)	DSI > 1/3: No 58 (87.9); RT 62 (91.2) LVSI+: No 42 (63.6); RT 57 (83.8)
Cao, 2021 [12]	Retrospective	861	No 85 (9.9) RT 283 (32.9) CCRT 493 (57.2)	IB 414 (48.1) IIA 447 (51.9)	NA	2–4 cm: No 19 (22.6); RT 56 (19.8); CCRT 135 (27.4) ≥4 cm: No 65 (77.4); RT 227 (80.2); CCRT 354 (72.0)	LVSI+, deep third, any: No 14 (16.5); RT 55 (19.4); CCRT 124 (25.2) LVSI+, middle third, ≥2 cm: No 14 (16.5); RT 58 (20.5); CCRT 127 (25.8) LVSI+, superficial third, ≥5 cm: No 2 (2.4); RT 3 (1.1); CCRT 3 (0.6) LVSI−, middle/deep third, ≥4 cm: No 55 (64.7); RT 167 (59.0); CCRT 239 (48.5)
Nasioudis, 2021 [13]	Retrospective	765	No 387 (50.6) RT 378 (49.4)	IB	SCC: No 271 (70.0); RT 253 (67.0) Non-SCC: No 116 (30.0); RT 125 (33.0)	2–4 cm: No 201 (51.9); RT 180 (47.6) ≥4 cm: No 186 (48.1); RT 198 (52.4)	LVSI+: No 239 (62.9); RT 263 (72.9)
Turkmen, 2022 [14]	Retrospective	134	No 67 (50.0) RT 67 (50.0)	IB	NA	NA	NA
Cibula, 2023 [3]	Retrospective	692	No 274 (39.6) RT 418 (60.4)	IB	SCC: No 192 (70.3); RT 299 (71.7) Non-SCC: No 82 (29.7); RT 119 (28.3)	2–3.99 cm: No 156 (56.9); RT 241 (57.7) ≥4 cm: No 118 (43.1); RT 177 (42.3)	DSI > 1/3: NA LVSI+: No 194 (77.3); RT 332 (81.2)
Taguchi, 2023 [15]	Retrospective	960	No 480 (50.0) CT or RT 480 (50.0)	IB	NA	NA	NA
Tuscharoenporn, 2023 [16]	Retrospective	219	No 111 (50.7) CCRT or RT 108 (49.3)	IB 194 (88.6) IIA 25 (11.4)	SCC: No 81 (73.0); Adj 76 (70.4) Non-SCC: No 30 (27.0); Adj 32 (29.6)	No 3.12 ± 1.37 Adj 3.16 ± 1.33	DSI: Middle1/3 No 14 (12.6); Adj 21 (19.4); Outer1/3 No 97 (87.4); Adj 87 (80.6) LVSI+: No 100 (90.1); Adj 99 (91.7)

**Table 2:** Clinical outcome reported by the selected studies.

Study	Median FUP (Months)	Recurrence (*n*)	Site of Recurrence	5 y OS (%)
Sedlis, 1999 [4]	120.0	No 39 (27.9) RT 21 (15.3)	Local: No 27 (69.2); RT 18 (85.7) Distant: No 10 (25.6); RT 3 (14.3)	No 78.6 RT 86.9
Rotman, 2006 [7]	240.0	No 43 (30.7) RT 24 (17.5)	Local: No 29 (67.4); RT 19 (79.2) Distant: No 12 (27.9); RT 4 (16.7)	No 71.4 RT 80.3
Sartori, 2007 [8]	79.0	No 31 (14.4) RT 17 (14.7)	Local: No 24 (77.4); RT 8 (47.1) Distant: No 7 (22.6); RT 9 (52.9)	NA
Ryu, 2009 [9]	44.6	No 6 (17.6) RT 4 (8.1) CRT 89 2 (2.2)	NA	No 94.1 RT 97.6 CRT 98.6
Cibula, 2018 [10]	89.8	No 8 (6.3) RT/CRT 12 (12.2)	Local: No 2 (25.0); RT 0 (0.0) Distant: No 6 (75.0); RT 12 (100)	No 92.9 RT/CRT 85.6
Akilli, 2020 [11]	61.1	No 13 (19.7) RT 15 (22.1)	Local: No 8 (61.5); RT 9 (60.0) Distant: No 5 (38.5); RT 6 (40.0)	NA
Cao, 2021 [12]	63.0	No 6 (6.8) RT 15 (5.3) CRT 13 (2.6)	Local: No 6 (60.0); RT 15 (36.6); CCRT 13 (24.5) Distant: No 3 (30.0); RT 25 (61.0); CCRT 36 (67.9)	NA
Nasioudis, 2021 [13]	45.0	NA	NA	No 87.1 RT 88.4
Turkmen, 2022 [14]	60.0	No 12 (17.9) RT 7 (10.4)	NA	NA
Cibula, 2023 [3]	55.2	No 44 (16.1) RT 84 (20.1)	Local: No 29 (65.9); RT 14 (16.7) Distant: No 37 (84.1); RT 47 (55.6)	No 92.4 RT 90.4
Taguchi, 2023 [15]	NA	No 57 (11.9) CT or RT 47 (9.8)	NA	No 94.2 CT or RT 94.0
Tuscharoenporn, 2023 [16]	76.1	No 13 (11.7) CRT or RT 9 (8.3)	NA	No 93.7 CRT or RT 89.8

## Data Availability

The authors confirm that the data supporting the findings of this study are available within the article.

## Supplementary Materials

The following supporting information can be downloaded at: https://www.mdpi.com/article/10.3390/cancers17081320/s1, Table S1: Newcastle-Ottawa comparability; Table S2: Jadad score RCT.
