# Supplementary material for: Adjuvant Therapy in “Intermediate-Risk” Early-Stage Cervical Cancer: To Treat or Not to Treat? Systematic Review and Meta-Analysis
Source: Cancers (Basel). 2025 Apr 14;17(8):1320. doi: 10.3390/cancers17081320 (PMC12026193; doi:10.3390/cancers17081320)
Supplement: Supplementary file 1 [file cancers-17-01320-s001.zip › cancers-3560427-supplementary.pdf]

Study	Selection	Comparability	Outcome	Overall
Sartori, 2007 [7]	★★★	★	★★★	7
Cibula, 2018 [9]	★★★★	★	★★★	8
Akilli, 2020 [10]	★★★★	★	★★★	8
Cao, 2021 [11]	★★★★	★★	★★★	9
Nasioudis, 2021 [12]	★★★	★	★★	6
Turkmen, 2022 [13]	★★★★	★	★★★	8
Cibula, 2023 [3]	★★★★	★	★★★	8
Taguchi, 2023 [14]	★★★★	★	★★★	8
Tuscharoenporn, 2023 [15]	★★★★	★	★★★	8

Table S1. Newcastle -Ottawa comparability

Study	Items								Total	Quality
	1	2	3	4	5	6	7	8		
Sedlis, 1999 [4]	1	0	0	0	1	1	1	1	5	High
Rotman, 2006 [6]	1	0	0	0	1	1	1	1	5	High

Table S2 Jadad score RCT
